# Soil phosphorus partitioning and bacterial communities vary with prolonged *Camellia oleifera* cultivation

**DOI:** 10.3389/fmicb.2026.1944090

**Published:** 2026-09-30

**Authors:** Yukun Lu, Zipei Luo, Jing Yang, Lichao Wu, Sheng Lu

**Affiliations:** 1 Key Laboratory of Soil and Water Conservation and Desertification Combating in Hunan Province, College of Forestry, Central South University of Forestry and Technology, Changsha, China; 2 Faculty of Civil and Environmental Engineering, Technion-Israel Institute of Technology, Haifa, Israel; 3 National Engineering Laboratory of Applied Technology for Forestry & Ecology in Southern China, Central South University of Forestry and Technology, Changsha, China

**Keywords:** bacterial community, *Camellia oleifera*, organic matter, phosphorus adsorption, phosphorus fractions, soil aggregates

## Abstract

**Introduction:**

*Camellia oleifera (C. oleifera)* is a valuable plant whose seeds produce tea seed oil, with potential applications in food, cosmetics, and agriculture. In the allitic soils of southern China, the complexity of soil phosphorus (P) fractions and influencing factors significantly restricts plant growth, especially for *C. oleifera.*

**Methods:**

This study examined soil samples from *C. oleifera.* plantations with cultivation periods of 2, 10, and 40 years, focusing on variations in P bioavailability among different soil aggregate sizes and the underlying regulatory mechanisms.

**Results:**

Soil P availability and its fractions changed significantly with cultivation period and aggregate size. Generally, as the cultivation period extended, soil available P (AP) declined by approximately 78.3%, while the root P accumulation factor increased by 657.91%, 687.01%, and 283.16% in large macroaggregates (LM), small macroaggregates (SM), and microaggregates (Mi), respectively. Maximum P adsorption capacity was highest in SM in the 2-year plantation but shifted to the Mi fraction in the 10- and 40-year plantations. Iron-bound phosphorus (Fe-P) was the predominant inorganic P fraction and showed a consistent distribution pattern across aggregate sizes. With increasing cultivation time, calcium-bound phosphorus (Ca-P) and occluded phosphorus (O-P) decreased, whereas aluminum-bound phosphorus (Al-P) increased in larger aggregates. AP and P fractions were negatively associated with bulk soil organic matter (SOM) content, while individual P fractions showed contrasting relationships with non-hydrophilic and hydrophilic SOM functional groups. Additionally, O-P, Al-P and Fe-P were positively correlated with bacterial ɑ-diversity in LM but negatively correlated in SM and Mi. Specific bacterial taxa were associated with different P forms.

**Discussion:**

Overall, the results show that aggregate specific P partitioning, SOM composition, P adsorption characteristics and bacterial community patterns are closely associated with soil P availability during prolonged *C. oleifera.* cultivation.

## Introduction

Phosphorus (P) is a vital nutrient for plant growth, yet it is characterized by low content and limited bioavailability, leading to severe P deficiency in the allitic soil regions, particularly in subtropical regions such as South China (Fu et al., 2020). P in soils is found in both organic and inorganic states, with plants primarily absorbing it in the inorganic forms (e.g., HPO_4_^2–^ and H_2_PO_4_^–^). The biotic factors that regulate P transformation significantly influence its availability to plants. For example, microbial activity and enzyme functions are crucial for the decomposition of organic P, which releases inorganic P that plants can utilize (DeForest and Moorhead, 2020). The soil’s mineral composition and the presence of metal ions like calcium (Ca), iron (Fe), and aluminum (Al) also play a critical role by binding with inorganic P, thereby reducing its mobility and availability (Qaswar et al., 2022). Allitic soils are distinguished by elevated concentrations of iron (Fe), aluminum (Al), and secondary clay minerals, along with advanced weathering, which collectively contribute to low P availability due to the interplay of various factors (Chakraborty et al., 2024). Consequently, changes in total P alone cannot adequately describe soil P limitation because available P depends largely on how P is partitioned among retained fractions. However, the primary factors reshaping the P partitioning in allitic soils require further systematic research to be fully understood.

*Camellia oleifera (C. oleifera)*, an economically valuable tree, is primarily grown for its seeds, which produce tea seed oil that is rich in oleic acid (Yang et al., 2016). In addition to its culinary applications, the oil is widely used in various industrial sectors, including the production of soaps, lubricants, paints, and as a rust-proofing agent (Quan et al., 2022). *C. oleifera* is predominantly cultivated in allitic soils and has a stable harvest period that can extend up to 80 years. Stand age can substantially alter aboveground biomass, SOM quantity and quality and soil microbial communities in perennial plantations. In *C. oleifera* plantations, microbial P limitation has been reported to intensify with increasing stand age, accompanied by changes in microbial community organization (Quan et al., 2022). Similar stand age associated changes in rhizosphere P availability and microbial diversity have also been reported (Qiao et al., 2021; Li et al., 2024). Soil P content is a key limiting factor for C. oleifera productivity, particularly under conditions of continuous harvesting (Liu et al., 2017). One of the main obstacles to enhancing plant growth in these soils is the low P utilization efficiency, which typically ranges between 10% and 20% (Liu et al., 2017; Meyer et al., 2021). Microorganisms play a critical role in several biogeochemical processes involved in the P cycle, including P uptake, release, and redistribution, thereby enhancing P availability by more than 20% (Pistocchi et al., 2018; Lv et al., 2025). However, these stand age studies have primarily focused on bulk or rhizosphere soil, leaving unresolved whether plantation age associated changes in P status are expressed differently among soil aggregate fractions.

Long-term cultivation of plants can accelerate soil weathering and increase the content of secondary clay minerals (Lu et al., 2018). Elevated levels of Fe/Al (hydr)oxides and secondary clay minerals increase P fixation by facilitating the formation of P-containing minerals (Meyer et al., 2021; Ichinose et al., 2025). Xiong et al. (2022) have found that allitic soils, which have a higher content of clay minerals, provide more sites for P adsorption compared to Fe/Al (hydr)oxides, with the P adsorption capacity of clay minerals being more than six times higher. Similarly, Herlihy and McGrath (2007) demonstrated a significant linear relationship between the content of clay minerals and soil available phosphorus (AP). Gérard (2016) demonstrated that, over more than 70 years, clay minerals, with more P-binding constituents, play a greater role in P adsorption than Fe/Al (hydr)oxides in experimental studies. The factors influencing P availability are complex, and the primary regulatory mechanisms may vary depending on the different planting periods of *C. oleifera*. Therefore, additional studies are required to explore the key factors and mechanisms that control soil P availability during different growth stages of *C. oleifera*.

Soil aggregate particle sizes provide insights into how different ecological niches influence SOM content and composition, microbial community structure and plant nutrient accumulation (Hu et al., 2025). Various sizes of soil aggregates contain differing amounts of SOM and clay minerals, which in turn affect P adsorption and its various fractions, such as Al-P, Fe-P, and Ca-P (He et al., 2021). The specific forms of P significantly impact its bioavailability to plants. Cui et al. (2025) have reported that inorganic labile P, organic labile P, and residual P levels tend to decrease with smaller soil aggregate sizes, whereas mineral-associated organic P is enriched in small aggregates ( < 0.25 mm). Research by Zhang et al. (2020) has shown a significant correlation between total phosphorus (TP) and Olsen P contents with soil organic carbon (SOC). Moreover, during long-term plant cultivation, P content and its fractions remain relatively stable within macro-aggregates. As an important physical property, soil aggregate composition varies significantly across different plant cultivation periods, subsequently affecting plant P uptake (PPU). For example, Wang et al. (2020) found macro-aggregates serve as key structural units for supplying soil P. Under nitrogen (N) fertilizer addition and mowing management, plant biomass and species richness are closely related to the available nutrients in macro-aggregates. Therefore, understanding the relationship between soil aggregate composition and P availability is crucial for optimizing nutrient management. Further research is needed to explore how aggregate sizes influence P cycling and PPU across different cultivation systems.

Previous studies have primarily investigated PPU under short term management interventions, such as mineral fertilization and manure application (Zhang et al., 2020). Recent studies have begun to reveal aggregate specific microbial regulation of soil P fractions under fertilization, mowing, and nitrogen addition (Cui et al., 2025; Li et al., 2025). These studies have demonstrated that soil aggregates can differentiate P fractions and their associated microbial communities. However, whether aggregate specific P partitioning varies systematically among plantation ages and how such variation is associated with bacterial community attributes, remains poorly understood in long lived woody plantations. In particular, it is unclear whether plantation age associated changes in P adsorption and SOM composition coincide with the redistribution of individual P fractions and whether these P changes are associated with bacterial community patterns across aggregate sizes. Addressing this knowledge gap is essential for identifying the physicochemical and biological factors that regulate soil P availability and PPU in long-term *C. oleifera* plantations.

Based on these observations, we hypothesized that older *C. oleifera* plantations would exhibit lower soil P availability and stronger P retention in macroaggregates as SOM composition and P adsorption characteristics change. We further hypothesized that these aggregate specific changes in P partitioning would be accompanied by corresponding shifts in bacterial community composition and diversity. The current study aimed to: (1) determine how soil AP and inorganic P partitioning vary among *C. oleifera* plantation ages and aggregate fractions; (2) evaluate the relationships of SOM content and functional groups with aggregate specific P partitioning; and (3) determine whether associations between bacterial community attributes and individual P fractions differ among aggregate sizes and identify the major physicochemical and biological factors associated with soil P availability. These objectives were designed to clarify how cultivation duration reshapes P partitioning and bacterial communities in soil aggregates, thereby improving our understanding of soil P dynamics in long-term *C. oleifera* plantations.

## Materials and methods

### Sampling location and experimental design

The study was carried out in Youxian County, located in Hunan Province, China (27° 03’ 45” N, 113° 24’ 31” E), an area known for its humid subtropical monsoon climate, with an average annual temperature of 17.8 °C and yearly precipitation of 1,410 mm. The experimental site is situated on hilly terrain with a slope of less than 15°. The predominant soil type in the C. oleifera plantation is allitic soil, classified as clay soil in terms of texture. Baseline soil nutrient values were recorded as a pH of 4.30, 13.23 g/kg of SOM, 0.77 g/kg of total nitrogen (TN), 1.05 g/kg of total phosphorus (TP) and 27.83 g/kg of total potassium (TK). These highly weathered acidic soils are naturally characterized by relatively low P availability, providing an appropriate background for examining changes in soil P partitioning and availability.

The study focused on the *C. oleifera* cultivar Xianglin No. 1, which is the most extensively cultivated variety in Hunan Province. To examine soil P transformation and PPU across different plant cultivation periods, three types of *C. oleifera* were selected, representing cultivation durations of 2 years, 10 years and 40 years. These stages correspond to the initial growth stage (Chi), high-yield stage (You) and decline stage (Old). To reduce environmental heterogeneity unrelated to plantation age, all plots were deliberately selected within the same relatively restricted geographic area. The selected plantations occurred under comparable climatic and topographic conditions, had the same general soil type and had similar management practices, with no fertilizer application. Information on sample plots was based on previous study (Lu et al., 2022). Therefore, restricting plot selection to this environmentally similar area was intended to minimize variation associated with soil type, topography, climate and management history.

For each cultivation period, three independent 20 m × 20 m plots were established (Chen et al., 2022). In each standard plot, five soil samples from the top 20-cm soil layer were collected and combined to form a composite sample. Accordingly, the three plots constituted the independent biological replicates for each plantation age class (*n* = 3), whereas the five within-plot soil samples were spatial subsamples. Approximately 1 kg of these composite soil samples were collected, stored in an icebox, and transported to laboratory within 8 h. The soil was divided into two parts. One part was air dried at room temperature for determination of soil physicochemical properties (soil aggregate, soil phosphorus fractions, soil organic matter and functional groups), and the other part was stored at −80 °C for microbial analysis.

### Soil aggregate composition

Soil water-stable aggregates were separated using a wet-sieving method modified from Elliott and Cambardella (1991). Air-dried soil samples were gently placed on a set of nested sieves with mesh sizes of 2 and 0.25 mm and slowly immersed in deionized water for 5 min to allow complete pre-wetting. The sieves were then gently moved vertically in water to separate the soil into three aggregate-size fractions: large macroaggregates ( > 2 mm; LM), small macroaggregates (0.25−2 mm; SM), and microaggregates ( < 0.25 mm; Mi). The aggregate fractions retained on each sieve and collected from the suspension were recovered separately and prepared for subsequent analyses of soil physicochemical properties and bacterial community composition.

### Phosphorus isothermal adsorption experiment

The P adsorption capacity of different soil aggregate fractions was assessed using a batch equilibrium adsorption experiment followed by Langmuir isotherm fitting, based on the method of Olsen and Watanabe (1957) with modifications. The soil samples were air-dried and passed through a 1-mm sieve. About 1.00 g of soil was weighed and transferred into a 50-mL centrifuge tube, then 25 mL of KH_2_PO_4_ solution with phosphorus concentrations of 0, 2, 5, 10, 15, 20, 30, 50, and 75 mg/L was added. To prevent microbial activity, three drops of chloroform were introduced into each solution. The centrifuge tubes were sealed and shaken at 200 rpm for 24 h at 25 °C in a dark environment to ensure adsorption equilibrium. After equilibration, the samples were centrifuged at 4,000 rpm for 10 min, and the P concentration of the supernatant was measured using a SmartChem automatic discontinuous chemical analyzer (WestCo Scientific Instruments, Brookfield, CT, USA). The P adsorbed by the soil was determined by measuring the difference in P concentration before and after equilibration. The maximum adsorption capacity was calculated by fitting the data to either the Langmuir adsorption isotherm models using Origin 2022 software. Each result represented the mean of three replicates.

### Soil P fraction measurement

Soil inorganic P fractions were determined using a modified sequential extraction procedure based on the Chang-Jackson fractionation framework, as applied to acidic red soils (Chang and Jackson, 1957b; Wang et al., 2014). The soil samples underwent extraction with a series of extractants in the following order: (1) 0.5 M ammonium fluoride (NH_4_F) was used to extract Al-P. (2) The samples were then subjected to extraction with a saturated NaCl solution twice, followed by 0.1 M sodium hydroxide-sodium carbonate (NaOH-Na_2_CO_3_) for Fe-P. (3) Next, the samples were continuously extracted with a saturated NaCl solution twice. Following this, sodium citrate (Na_3_C_6_H_5_O_7_), sodium dithionate (Na_2_S_2_O_4_), and NaOH were used sequentially, after which the samples were boiled with sulfuric acid (H_2_SO_4_) and placed in a water bath to extract occluded phosphorus (O-P). (4) The remaining residue was further extracted with a saturated NaCl solution twice, followed by a 1/2 H_2_SO_4_ solution for Ca-P. The supernatants from each extraction step were centrifuged at 5,000 rpm for 15 min and analyzed using the ascorbic acid-molybdenum blue method (Chang and Jackson, 1957b).

The total phosphorus (TP) concentration was quantified using sodium hydroxide fusion followed by molybdenum-antimony colorimetry, while the available phosphorus (AP) concentration was measured using the Mehlich 3 extraction method (Mehlich, 1984). Mehlich-3 was selected because it is applicable across a broad range of soil conditions and is well established as an index of soil-test P in acidic to neutral soils (Burt, 2011; Matcham et al., 2023). The measurement method of root TP follows Mori et al. (2016). A 0.5 g root sample was digested using a mixed solution of nitric acid, perchloric acid, and sulfuric acid in a ratio of 3:1:1. The Pi concentration in the extract was then determined using the vanadomolybdate colorimetric method.

### Soil organic matter and functional groups

The functional groups of soil organic matter (SOM) were examined using a Fourier transform infrared (FTIR) spectrometer. The soil sample was dried at 60°C for 8 h to the analysis to minimize interference of the hydroxyl group in the infrared spectrum. Spectroscopic grade KBr was ground to a particle size of < 2 μm using an agate mortar, thoroughly mixed with the soil at a soil:KBr mass ratio of 1:100, and then pressed into pellets for 5 min at 75 kPa. The resulting spectrum was scanned and measured using the FTIR spectrometer.

### Soil enzyme activity

Catalase (CAT) activity was assessed using the potassium permanganate titration method as described Stepniewska et al. (2009). About 5 g of soil was treated with 25 mL of 3% H_2_O_2_ and incubated at 4 °C for 30 min to allow complete reaction. The reaction was stopped by adding 25 mL of 2 mol/L H_2_SO_4_, and the resulting mixture was titrated using a 0.02 mol/L potassium permanganate solution.

Sucrase (SuCr) activity was measured using the 3, 5-dinitrosalicylic acid (DNS) colorimetric method as described by Schinner and von Mersi (1990). A total of 5 g of soil was mixed with 15 mL of 8% sucrose solution, 5 mL of pH 5.5 phosphoric acid buffer, and five drops of toluene. The mixture was shaken and incubated in an incubator at 37°C for 24 h. Then the solution was filtered and about 1 mL of the filtrate was used to suction and reacted with 3 mL of DNS (3, 5-dinitrosalicylic acid). Lastly, the sample was diluted to a final volume of 50 mL with distilled water and analyzed colorimetrically at 508 nm using a spectrophotometer.

Urease (Ure) activity was assessed using the sodium phenol-sodium hypochlorite colorimetric method described by May and Douglas (1976). Approximately 5 g of soil was mixed with 1 mL of toluene, 10 mL of a 10% urea solution, and 20 mL of a pH 6.7 citrate buffer, then incubated at 37 °C for 24 h. After incubation, 1 mL of the filtrate was combined with 4 mL of sodium phenol solution and 3 mL of sodium hypochlorite solution, and the absorbance was measured at 578 nm using a spectrophotometer.

Acid phosphatase (ACP) activity was measured using the colorimetric method outlined by Nannipieri et al. (2011). About 5 g of soil was incubated with 2.5 mL of toluene and 20 mL of 0.5% disodium phenyl phosphate at 37 °C for 24 h. After incubation, 100 mL of 0.3% aluminum sulfate solution was added, followed by the addition of 3 mL of the filtrate mixed with boric acid buffer. The absorbance was then measured at 660 nm using a spectrophotometer.

### 16 S rRNA gene high-throughput sequencing

The soil bacterial community composition was analyzed through 16S rRNA gene high-throughput sequencing. Soil DNA was extracted using the E.Z.N.A. Stool DNA Kit (Omega, USA), and the V4–V5 region of the 16S rRNA gene was amplified with primers F (5’-GTGCCAGCMGCCGCGG-3’) and R (5’-CCGTCAATTCMTTTRAGTTT-3’). PCR products were analyzed by 2% agarose gel electrophoresis. The amplicon library was sequenced using a NovaSeq PE250 platform, and quality control was performed with an Agilent 2100 Bioanalyzer (Agilent, USA) and the Illumina Library Quantification Kit (Kapa Biosciences, Woburn, MA, USA). Raw reads were processed with fqtrim (v0.94) to obtain high-quality clean sequences. Reads with expected errors exceeding 0.2 were removed during quality filtering. Paired-end reads were subsequently processed using the DADA2 pipeline, including dereplication, error-model-based denoising, and paired-end read merging. Chimeric sequences were identified and removed using Vsearch (v2.3.4). The resulting high-quality sequences were clustered into operational taxonomic units (OTUs) at a sequence similarity threshold of 97% using Vsearch (v2.3.4). Singleton OTUs were removed before downstream analyses. After quality filtering, denoising, chimera removal and OTU clustering, a total of 8529 OTUs were retained. Across the sequencing dataset, a total of 1,325,997 high-quality valid sequences were obtained, with 61,377–81,485 valid sequences per sample. Most valid sequences (1,322,679; 99.75%) were 300–400 bp in length. To minimize potential biases arising from unequal sequencing depth among samples, the OTU table was rarefied to 27,679 sequences per sample prior to α- and β-diversity analyses. Rarefaction curves based on observed OTUs showed a clear tendency toward asymptotes at this sequencing depth, indicating that the sequencing effort captured the majority of bacterial community diversity present in the samples (Supplementary Figure 3). Taxonomic assignment of representative OTU sequences was performed against the SILVA database (Release 132) using a confidence threshold of 0.7. Subsequent diversity analyses and visualizations were conducted in R (version 4.3.1). The raw sequencing data are available in the NCBI Sequence Read Archive (SRA) database under accession number SRP332322. The 16S rRNA sequencing dataset analyzed in the present study is the same dataset as that reported in our previous study (Lijun et al., 2023), and no additional 16S rRNA sequencing was conducted for the present study. The previous study primarily used this dataset to characterize bacterial community composition, diversity, and community assembly processes. In the present study, this dataset was analyzed specifically to evaluate aggregate specific relationships between bacterial community attributes and soil P fractions and was further integrated with P adsorption and SOM properties to investigate the physicochemical and biological factors associated with soil P availability.

## Statistical analysis

All data were initially processed using Microsoft Office Excel (2003) and further analyzed with SPSS software (version 19.0). For most variables presented in the main figures, differences among plantation-age classes were evaluated separately within each aggregate fraction (LM, SM, and Mi) using one-way analysis of variance (ANOVA), followed by Tukey’s HSD test for multiple comparisons when significant differences were detected (*p* < 0.05). For key soil properties, including TP, AP, SOM, and pH, the effects of plantation age, aggregate size, and their interaction were further evaluated using a two-way repeated-measures ANOVA. Plantation age was treated as a between-plot factor, whereas aggregate size was treated as a within-plot factor because LM, SM, and Mi were separated from the same plot-level composite soil sample. The plot was considered the independent biological replicate, with three plots per plantation-age class (*n* = 3). The P bioaccumulation factor (BAF) was calculated as follows:


BAF=TP/r⁢o⁢o⁢tAPs⁢o⁢i⁢l

where TP_*root*_ is the total P concentration in root tissue (mg/kg dry mass) and AP_*soil*_ is Mehlich-3-extractable soil P (mg/kg soil). In this study, BAF was used to quantify root P enrichment relative to the soil available-P pool.

Figures 1–6 were generated using Python 3.9. Differences in bacterial community composition were evaluated using principal coordinate analysis based on Bray-Curtis dissimilarities. The effects of plantation age on bacterial community composition within each aggregate fraction were tested using permutational multivariate analysis of variance with 999 permutations using the adonis function in vegan package in R. Pearson correlation analysis was used to examine associations between bacterial α-diversity indices and soil properties. Mantel tests were used to assess relationships between P fractions and physicochemical and biological variables. OTUs of the microbial community that exhibited significant responses to environmental factors were identified, with threshold values determined using Threshold Indicator Taxa Analysis (TITAN) via the “TITAN2” package in R (Baker and King, 2010). Partial least squares path modeling (PLS-PM) was used to evaluate the direct and indirect relationships among five conceptual components: plantation age, soil chemical properties, soil physical properties, biological properties and soil P availability. Soil chemical properties were represented by TP, O-P, Al-P, Fe-P, Ca-P, pH, AN, AK, SOM, C/N, -OH and -CH; physical properties by mean weight diameter (MWD), water-stable aggregate coefficient (kctp), clay%, silt%, Langmuir maximum adsorption capacity (Q_*m*_); biological properties by OTUs, ACP, Ure, SuCr and CAT; and soil P availability by AP. All continuous variables were standardized before model fitting. The hypothesized path structure was specified *a priori* according to the conceptual relationships among plantation age, soil properties, and P availability. PLS-PM was performed using the “plspm” package in R. The significance of path coefficients was evaluated by bootstrap resampling with 5000 iterations and 95% bootstrap confidence intervals were calculated. Model performance was assessed using the coefficient of determination (R^2^) for endogenous latent variables and the global goodness-of-fit (GoF) index. The overall model fit was classified as weak, moderate or strong based on thresholds of 0.1, 0.25, and 0.36 according to GoF (Wetzels et al., 2009; Lu et al., 2026a).

**FIGURE 1:**
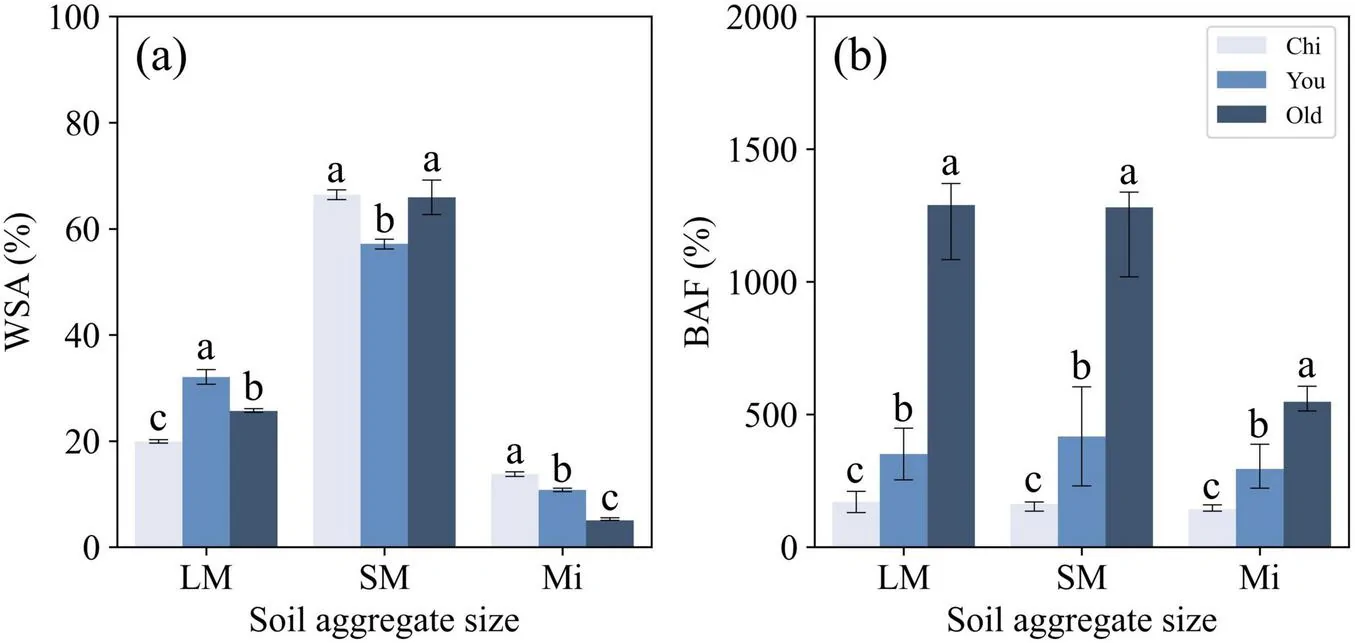
The distribution of soil water-stable aggregates (a) and root P bioaccumulation factor (BAF; TP_root_/AP_soil_) (b) across different *C. oleifera* cultivation periods. The cultivation periods were classified as initial growth stage (Chi), high-yield stage (You) and decline stage (Old). Soil aggregate sizes are categorized as follows: LM, large macroaggregate ( > 2 mm); SM, small macroaggregate (0.25-2 mm); and Mi, microaggregate ( < 0.25 mm). Significant differences among cultivation periods were labeled by different letters (p < 0.05).

**FIGURE 2:**
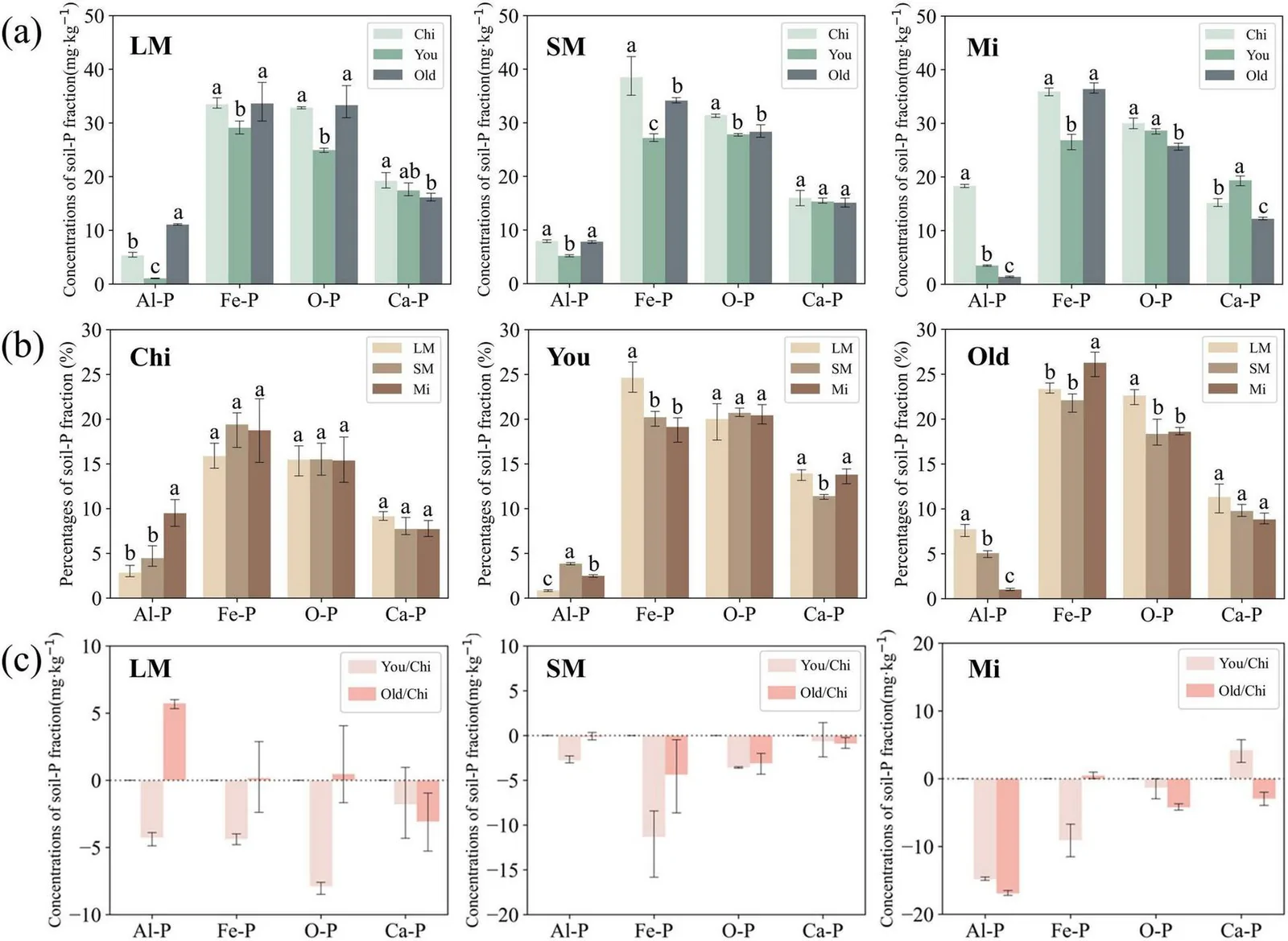
The concentration of soil P fractions **(a)**, the percentage of each kind of P to TP content **(b)**, and the variation of P fractions between different cultivation periods **(c)** were measured in three kinds of soil aggregates. The P fractions analyzed encompassed Al-P, Fe-P, O-P, and Ca-P. You/Chi represents the difference between You and Chi, and Old/Chi represents the difference between Old and Chi. Statistical significance among the cultivation periods is denoted by different letters (*p* < 0.05).

**FIGURE 3:**
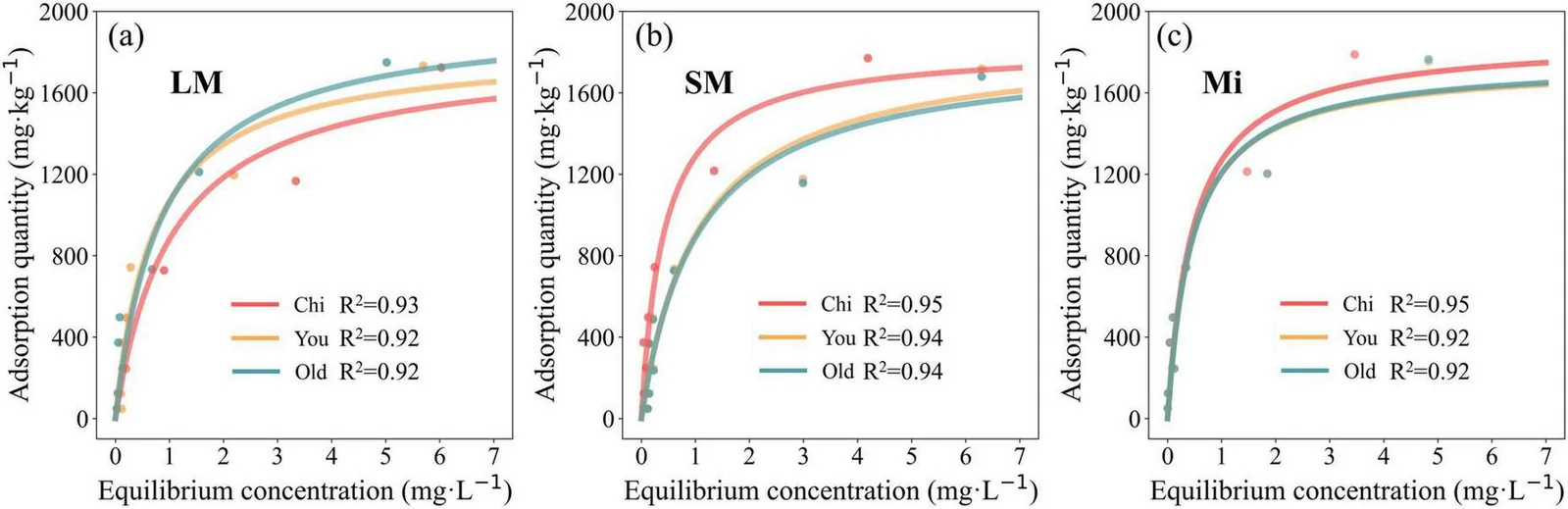
The P isothermal adsorption curves of different soil aggregates during different *C. oleifera* cultivation periods: **(a)** LM, **(b)** SM, and **(c)** Mi. R^2^ indicates the coefficient of determination for the Langmuir fit.

**FIGURE 4:**
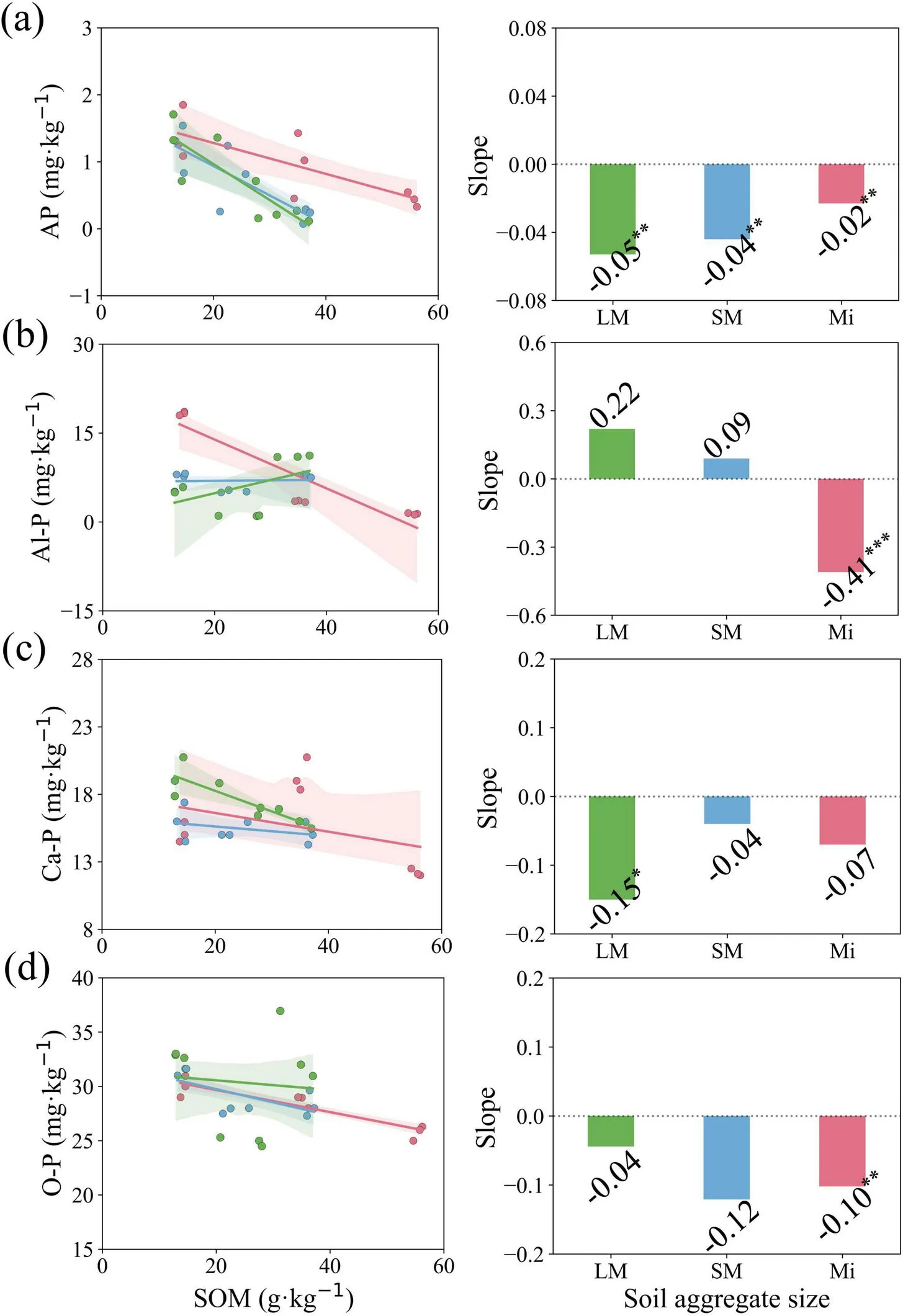
The linear relationships between SOM and **(a)** AP, **(b)** Al-P, **(c)** Ca-P, and **(d)** O-P content. The shaded areas around the lines represent the 95% confidence interval. *p < 0.05; **p < 0.01; ***p < 0.001 based on linear regression. The bars represent the slopes of the relationships between P fractions and SOM in different aggregate fractions, with the colors corresponding to the regression lines.

**FIGURE 5:**
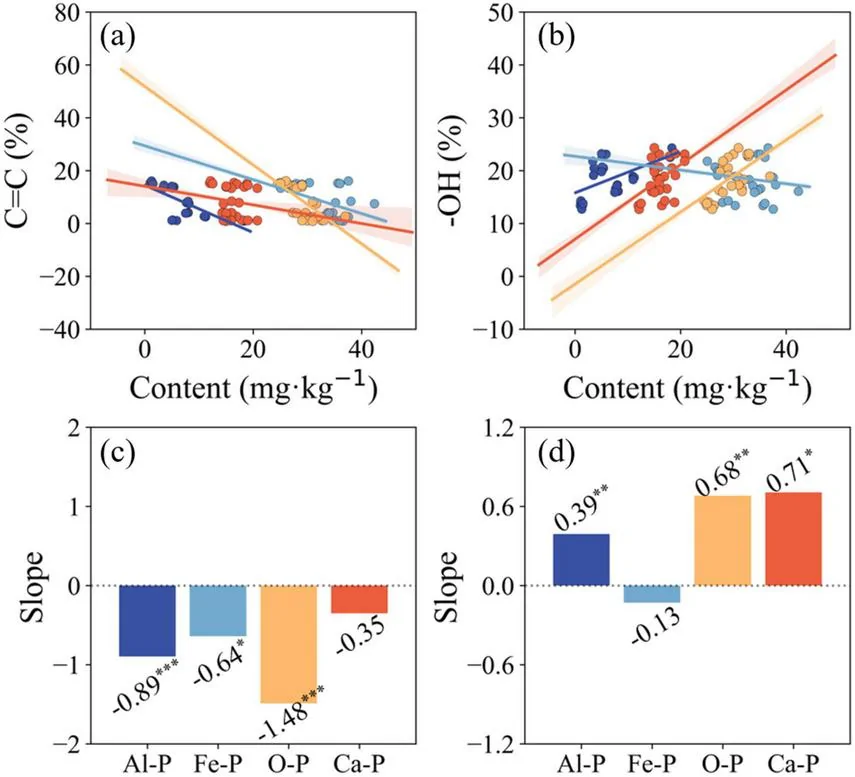
The linear relationship between the abundances of soil functional groups (C = C, -OH) and concentration of different P fractions. The shaded areas around the lines represent the 95% confidence interval. *p < 0.05; **p < 0.01; ***p < 0.001 based on linear regression. The bars **(c,d)** were represented by slopes of the lines in panels **(a,b)**, with the colors corresponding to the lines.

**FIGURE 6:**
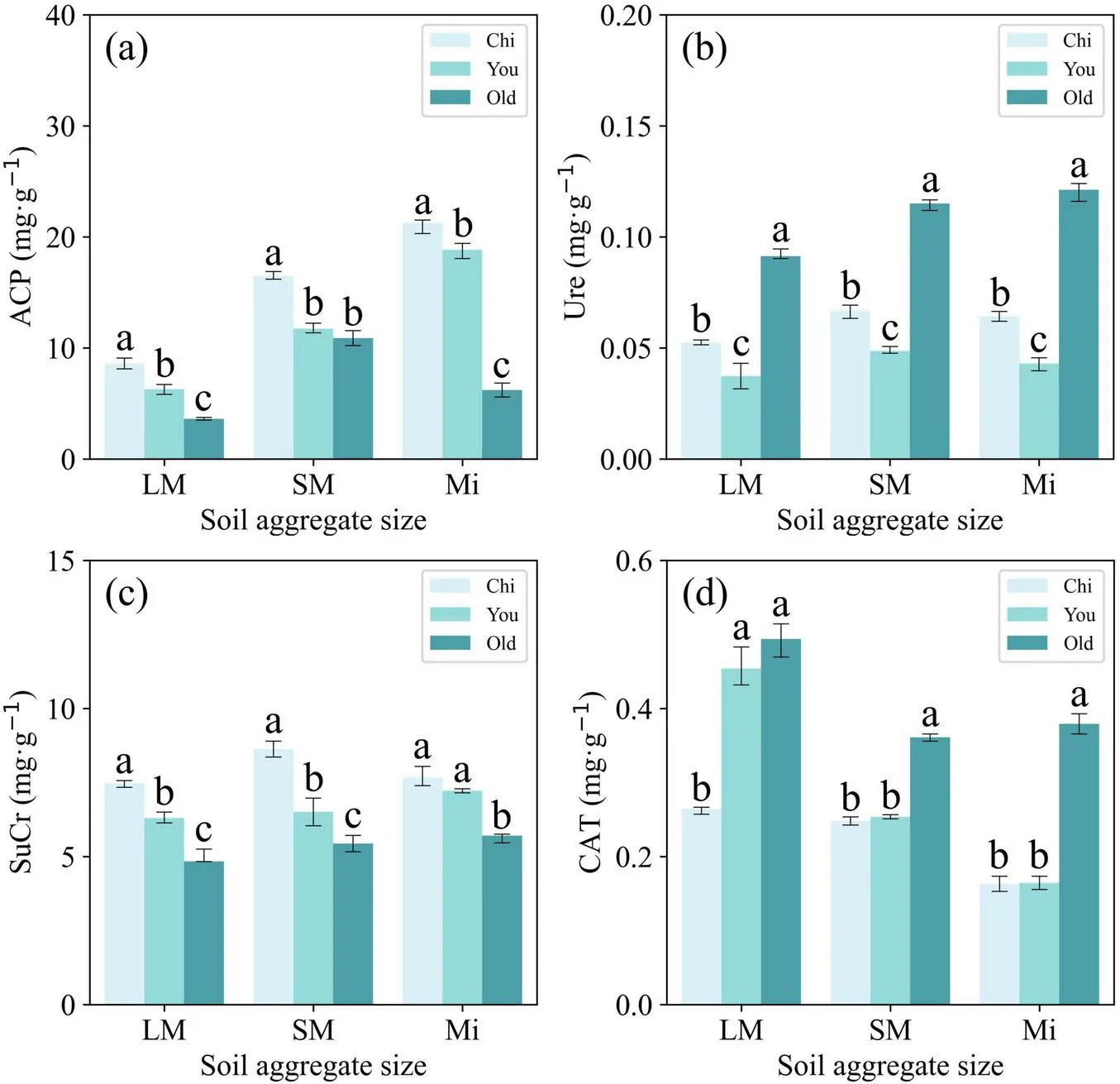
Variations in soil enzyme activities across different soil aggregate fractions and *Camellia oleifera* cultivation periods: **(a)** acid phosphatase activity (ACP), **(b)** urease activity (Ure), **(c)** sucrase activity (SuCr), and **(d)** catalase activity (CAT). Different lowercase letters indicate significant differences among cultivation periods within the same soil aggregate fraction (*p* < 0.05).

## Results

### P bioaccumulation within different soil water-stable aggregates

Long-term cultivation of C. oleifera significantly influenced the composition of soil water-stable aggregates and the root P bioaccumulation capacity (Figure 1). SM accounted for nearly 60% of the soil water stable aggregates, followed by LM and Mi (Figure 1a). The BAF significantly increased across all three particle size classes as the age of the C. oleifera plantation progressed, with the most notable rise observed in LM. Conversely, in Old group, P bioaccumulation in Mi was significantly smaller than that in LM and SM (Figure 1b).

### Distribution of available P and inorganic P fractions in soil aggregates

AP declined consistently with plantation age across all aggregate fractions, with decreasing by 78.3% from Chi to Old, whereas differences among aggregate sizes were not significant (Supplementary Table 1). Among the operationally defined inorganic P fractions, Fe-P and O-P were predominant, followed by Ca-P and Al-P (Figure 2a). Fe-P content was significantly lower in You than in Chi and Old, by 22.98% and 20.22%, respectively; nevertheless, Fe-P remained the dominant inorganic P fraction across the three aggregate size classes. O-P content was 11.49% and 14.07% lower in SM and Mi, respectively, than in LM. In contrast, the aggregate distribution of Al-P differed among plantation-age classes, with relatively higher Al-P occurring in Mi of Chi, SM of You, and LM of Old soils (Figures 2b,c).

### P adsorption characteristic of soil aggregates

P adsorption capacity in *C. oleifera* soils varied significantly across different plantation periods and soil aggregate sizes (Figure 3). The Q_*m*_ showed distinct aggregate specific patterns. In Chi soils, Q_*m*_ was highest in SM (4362.99 mg/kg), followed by Mi (3954.94 mg/kg) and LM (1716.21 mg/kg). In contrast, Mi showed the highest Q_*m*_ in both You (3892.15 mg/kg) and Old (3846.69 mg/kg), followed by LM and SM. Relative to Chi, Q_*m*_ in Old increased by 34.42% in LM but decreased by 59.77% and 2.74% in SM and Mi, respectively. The Langmuir adsorption constant (K_*L*_) and maximum buffer capacity (MBC) also varied among aggregate fractions, but neither showed a consistent aggregate size pattern across all plantation age classes (Supplementary Table 2).

### Relationship between soil P fractions and SOM

Linear regression analysis was conducted to explore the relationship between soil AP content and other soil chemical properties. Soil AP was significantly negatively associated with SOM content (Figure 4 and Supplementary Figure 1), with the steepest negative regression slope observed in LM (Figure 4a). Several inorganic P fractions were also negatively associated with SOM content (Figures 4b-d). Among these relationships, Al-P showed the steepest negative slope in Mi (Figure 4b), whereas Ca-P showed the steepest negative slope in LM (Figure 4c).

The degree of SOM aromatization increased with rising SOM content (Supplementary Figure 2 and Supplementary Table 3). All four types of soil P fractions were negatively correlated with the increasing presence of non-hydrophilic groups, such as C = C (Figures 5a, c). In contrast, hydrophilic groups (such as -OH) in the soil were positively correlated with the contents of Al-P, O-P, and Ca-P (Figures 5b, d). The negative relationships with non-hydrophilic groups were stronger for P fractions with relatively higher plant bioavailability (Figure 5c).

### Variations in soil enzyme activities

Soil enzyme activities varied significantly across different *C. oleifera* plantation periods and soil aggregate sizes (Figure 6). ACP activity decreased significantly with increasing plantation age of *C. oleifera* and increased as soil aggregate size decreased (Figure 6a). Ure activity followed the order of Old > Chi > You (Figure 6b). SuCr activity showed a marked decline with longer plantation periods of *C. oleifera* (Figure 6c). CAT activity was higher in Old soils compared to the other treatments (Figure 6d).

### Bacterial community patterns and associations with soil P fractions

Principal coordinate analysis revealed clear separation of bacterial communities among the three *C. oleifera* plantation periods within each soil aggregate fraction (Figure 7). The first two principal coordinate axes explained 83.24%, 88.53% and 81.02% of the total variation in bacterial community composition in LM, SM and Mi, respectively. Bacterial α-diversity differed among plantation ages, with the magnitude and direction of variation depending on the diversity index and aggregate fraction (Supplementary Figure 4). Bacterial α-diversity indices were significantly associated with several soil physicochemical and biological properties, including TN, AN, NH_4_^+^, O-P, Al-P, and Ure in LM; with SOM, AK, AP, O-P, Al-P, ACP and SuCr in SM; SOM, AK, AP, O-P, Al-P, ACP, Ure, SuCr, and CAT in Mi (Figure 7). Soil bacterial diversity was positively correlated with O-P, Al-P, and Fe-P contents in LM aggregates, but negatively correlated with these fractions in SM and Mi aggregates.

**FIGURE 7:**
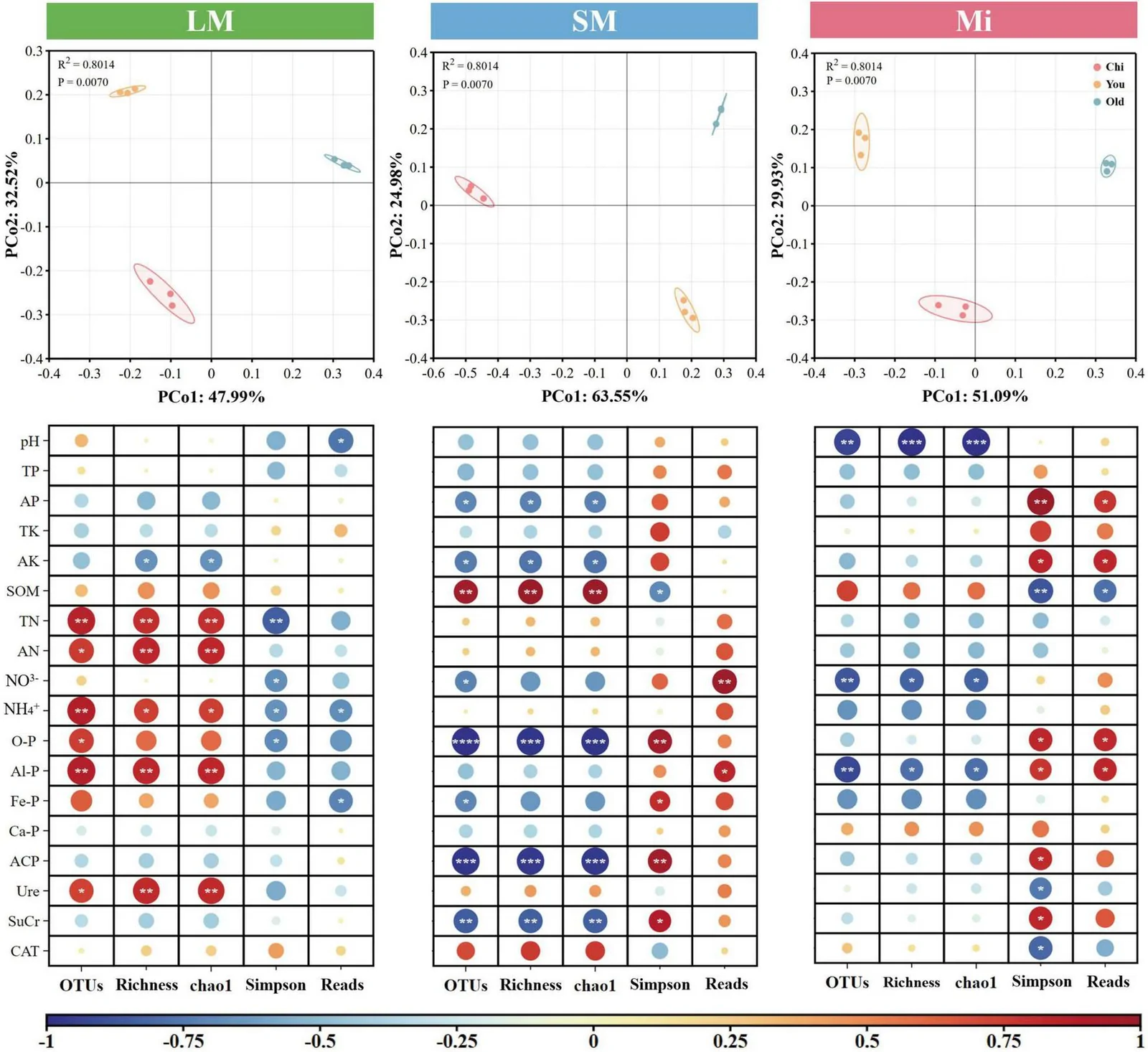
Bacterial community composition and its relationships with soil physicochemical properties across soil aggregate fractions and *Camellia oleifera* cultivation periods. The upper panels show principal coordinate analysis (PCoA) based on Bray–Curtis dissimilarities for bacterial communities in large macroaggregates (LM, > 2 mm), small macroaggregates (SM, 0.25–2 mm), and microaggregates (Mi, < 0.25 mm). Points represent individual samples, and ellipses indicate the distribution of samples within each cultivation period. Chi, You, and Old represent the initial growth, high-yield, and decline stages of *C. oleifera* plantations, respectively. The reported R^2^ and *p*-values were obtained from PERMANOVA. The lower panels show Pearson correlations between bacterial α-diversity indices and soil properties. Circle color indicates the direction and magnitude of the correlation coefficient, with blue representing negative correlations and red representing positive correlations; circle size is proportional to the absolute correlation coefficient. Asterisks indicate significant correlations: *p < 0.05, **p < 0.01, ***p < 0.001, and ****p < 0.0001. TP, total phosphorus; AP, available phosphorus; TK, total potassium; AK, available potassium; SOM, soil organic matter; TN, total nitrogen; AN, available nitrogen; NO_3_^–^, nitrate nitrogen; NH_4_^+^, ammonium nitrogen; O-P, occluded phosphorus; Al-P, aluminum-bound phosphorus; Fe-P, iron-bound phosphorus; Ca-P, calcium-bound phosphorus; ACP, acid phosphatase activity; Ure, urease activity; SuCr, sucrase activity; CAT, catalase activity; OTUs, operational taxonomic units.

TITAN analysis was used to investigate the relationship between soil microbial niche and P fractions (Figure 8). We found that for Fe-P and O-P, indicator microorganisms showed significant z+ and z- with increasing content. However, regarding Ca-P, *Acidobacteria*, *Patescibacteria* and *Proteobacteria* were identified as indicator phyla showing significant abundance responses along the Ca-P gradient. Conversely, *Proteobacteria*, *Verrucomicrobia* and *Chloroflexi* showed stronger abundance responses to the gradients of the other P fractions, indicating close taxon specific associations with these P pools. This differential participation underscores the intricate microbial interactions and niche specializations within soil microbial communities that govern P dynamics.

**FIGURE 8:**
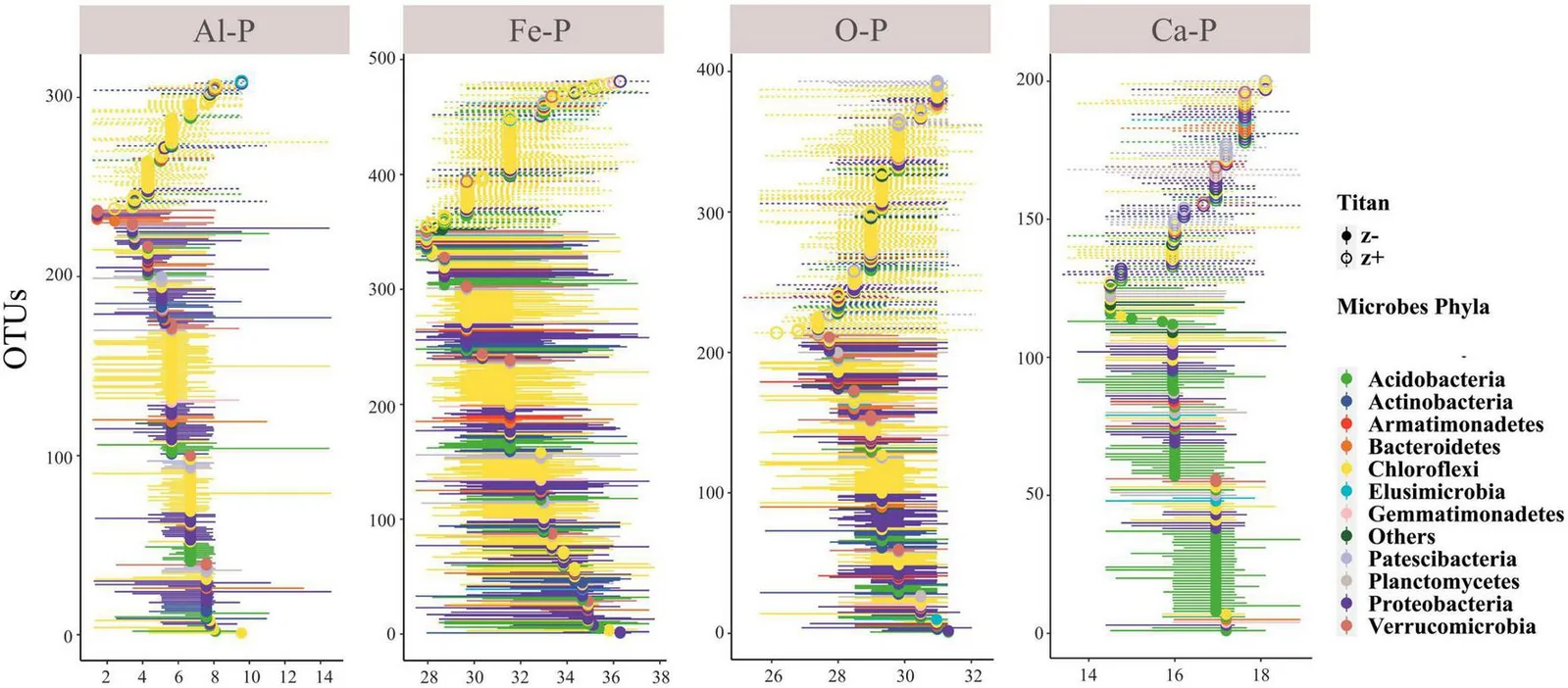
The relation of alteration in microbial OTU abundance to soil P fractions content is presented by using the TITAN. Each OTU is represented with subordinate phyla indicated by different colors, with filled symbols denoting a decline in abundance (z-) and open symbols indicating an increase in abundance (z+).

### Correlation between soil properties and P availability

The Mantel test revealed that soil P fractions exhibited varying degrees of responsiveness to different soil physicochemical and biological properties (Figure 9a). PLS-PM was employed to identify the most influential factors affecting soil P availability. The results showed that C. oleifera cultivation negatively impacted soil chemical properties (R^2^ = 0.80, *p* < 0.001) but had a significant positive effect on both soil physical properties (R^2^ = 0.93, *p* < 0.001) and biological properties (R^2^ = 0.81, *p* < 0.001). Among these, soil chemical properties played the most critical role in regulating soil P availability (λ = 0.85), followed by physical properties (λ = 0.74) and biological properties (λ = 0.70) (Figure 9b).

**FIGURE 9:**
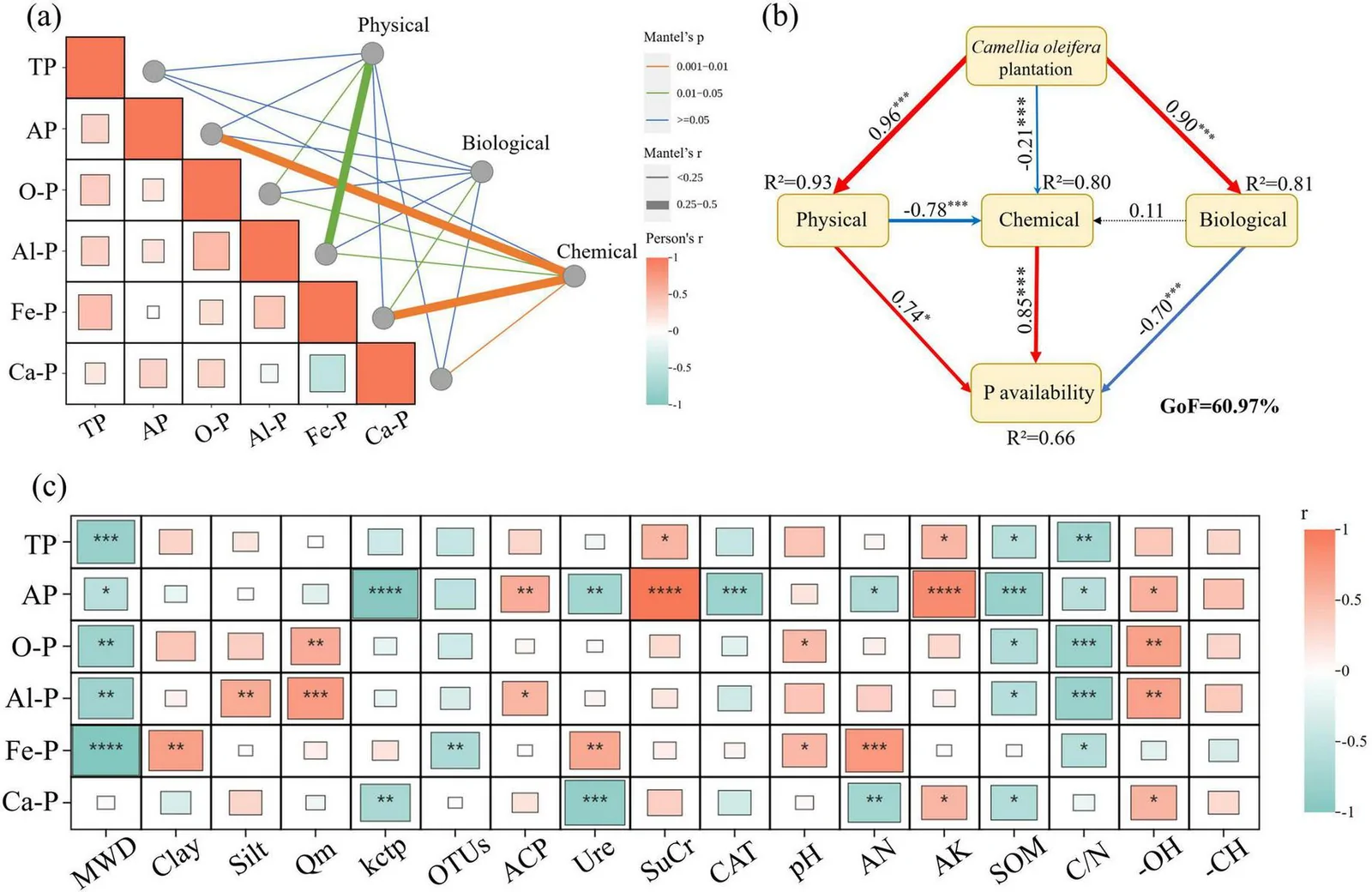
Relationships between soil P fractions and soil physicochemical and biological properties. **(a)** Mantel tests showing the associations between soil P fractions and environmental variables. Line width represents the strength of the Mantel correlation, and line style indicates statistical significance. **(b)** Partial least squares path model (PLS-PM) showing the relationships among the defined latent variables. Numbers on the arrows represent standardized path coefficients, and line width is proportional to the magnitude of the path coefficient. Solid lines indicate significant paths. R^2^ values indicate the proportion of variance explained for endogenous latent variables. **(c)** Pearson correlation coefficients among the measured soil properties. Asterisks indicate statistical significance: *p < 0.05, **p < 0.01, ***p < 0.001, and ****p < 0.0001.

Within the category of chemical properties, SOM content, C/N ratio and SOM functional groups emerged as the most critical indices influencing soil P availability. Key physical factors affecting soil P availability included the adsorption capacity of soil water-stable aggregates and soil texture. Biological properties, including bacterial community attributes and soil enzyme activities, were also significantly associated with soil P availability in the PLS-PM (Figure 9c).

## Discussion

### Activity and redistribution of soil P fractions in aggregates

Soil AP content significantly decreased by 78.29%from Chi to Old, while the BAF showed a substantial increase (Figure 1 and Supplementary Table 1). The marked decline in AP indicates increasing soil P scarcity in Old. Qiao et al. (2021) have also reported significant P limitations associated with increasing *C. oleifera* stand age through enzymatic stoichiometry analysis. The BAF increased more strongly in LM (657.91%) and SM (687.01%) than in Mi (283.16%), indicating progressively greater root P enrichment relative to the soil AP associated with these aggregate fractions. This ratio integrates plant P status with the size of the extractable soil P pool. Therefore, a higher BAF indicates that root tissue maintained greater P enrichment under lower soil P availability. Previous studies have indicated that the distribution of Pi is influenced by aggregate size (Wu et al., 2018). P fractions also exhibited distinct distribution patterns across different aggregates, highlighting the importance of understanding P dynamics within various aggregates to enhance PPU and promote *C. oleifera* productivity.

Fe-P was identified as the most abundant P fraction, displaying a consistent distribution pattern across different aggregates throughout the *C. oleifera* plantation periods.. Previous studies have suggested that Fe-P can contribute to AP pools (Lu et al., 2026b). And its utilization may vary relatively little among aggregate fractions under some soil conditions (Li et al., 2016; Wu et al., 2018). In contrast, Al-P was less abundant, showing enrichment in Mi for Chi soils and shifting to SM and LM in You and Old soils, respectively (Figure 2b). These findings were consistent with those of Cui et al. (2019), who have reported a gradual decrease in Al-P content with increasing aggregate size. The accumulation of Al-P in Mi in Chi soils (Figure 3c) could be attributed to the larger specific surface area of these aggregates (Li et al., 2016). However, there was a greater accumulation in SM or LM aggregates following *C. oleifera* cultivation (Figures 3a,b), underscoring the importance of SOM composition (Ahmed et al., 2024). In soils, the relative contribution of Pi fractions to crop availability typically follows the order: Al-P > Fe-P > Ca-P > O-P (Yang et al., 2012). Fe-P and Al-P are regarded as moderately available P forms; they play crucial roles in long-term soil P transformation and serve as buffers for readily AP in agroecosystems (Cui et al., 2019). Understanding the transformation mechanisms of Al-P and Fe-P within different aggregates is essential for enhancing P availability in soils.

### Soil P transformation mechanism and driving factors

To elucidate the mechanisms of P transformation and enhance P bioavailability, we conducted a systematic analysis of soil physicochemical and biological properties and their correlations with various P fractions (Figure 9). In Chi soils, BAF was relatively low and remained consistent across different aggregate types, indicating similar root P enrichment relative to the soil AP associated with LM, SM and Mi (Figure 1b). No significant differences were observed among the different P fractions, except for Al-P (Figure 2b). The soil P adsorption capacity increased with decreasing aggregate size (Figure 3 and Supplementary Table 2), which could be attributed to the higher specific surface area and greater adsorption capacity of Mi (Hunt et al., 2007; He et al., 2021). Additionally, in acidic conditions, H^+^ ions can protonate the surface groups of iron and aluminum oxides, resulting in increased P adsorption in fine-grained aggregates (Jin et al., 2006). In our study, AP was significantly negative correlated with bulk SOM content (Figure 4), and positively correlated with ACP (Figure 9) and the abundance of *Proteobacteria*, *Verrucomicrobia* and *Elusimicrobia* (Figure 8). These findings suggested that SOM, AP, aggregate-associated adsorption characteristics and biological properties covaried across the plantation-age gradient, suggesting that the observed AP pattern reflects the combined effects of multiple physicochemical and biological processes. However, this field scale relationship between bulk SOM and AP is not equivalent to the competitive sorption between specific reactive organic compounds and phosphate at mineral surfaces.

The aggregate distribution of P adsorption characteristics differed among plantation age classes (Figure 3). Notably, Q_*m*_ increased in LM but declined markedly in SM from Chi to Old, while remaining relatively stable in Mi, indicating a shift in P adsorption capacity from SM toward larger aggregates with plantation age (Supplementary Table 2). In the Old treatment, SOM content and microbial properties played a more crucial role in regulating soil P bioavailability (Figure 9). Specific reactive organic compounds can adsorb onto clay and Fe/Al oxide surfaces and compete with phosphate for sorption sites, although the magnitude and direction of these interactions depend on organic matter composition, mineralogy, P species and reaction conditions (Guppy et al., 2005; Spohn et al., 2022; Ahmed et al., 2024). Therefore, competitive sorption observed for particular organic compounds cannot be directly extrapolated to the relationship between bulk SOM concentration and soil AP under field conditions.Long-term crop cultivation increases the degree of humification and the stability of SOM (Deng et al., 2019; Omar et al., 2020). This shift in SOM composition provides an important context for the negative relationship between bulk SOM and soil P, because an increase in total SOM does not necessarily represent an increase in reactive organic ligands capable of competing with phosphate.

Notably, hydroxyl (-OH) groups can chelate multivalent metal ions such as Al^3 +^ (Stevenson, 1994), which can slow down the transformation of L-Pi forms that are weakly adsorbed by the soil. SOM functional groups have a stronger impact on P fractions with higher bioavailability (Figure 5). Hydrophilic groups, such as carboxyl domains with multiple negative charges, can restructure stable phosphate minerals (Yang et al., 2019). Meanwhile, non-hydrophilic phenolic groups primarily form complexes with di- and polyvalent cations (Kovar and Claassen, 2005). Therefore, bulk SOM content alone is insufficient to explain P availability and the chemical composition of SOM should be considered when interpreting its interactions with soil P.

The contents of Fe-P, Al-P, and O-P were significantly positively correlated with bacterial OTUs, species richness (Figure 7) and the abundance of dominant microbial communities, such as *Chloroflexi, Proteobacteria, Verrucomicrobia* and *Elusimicrobia* (Figure 8, Supplementary Figure 5). Previous studies have reported that some members of *Proteobacteria* possess traits associated with phosphate solubilization and P cycling (Bongoua-Devisme et al., 2024). *Chloroflexi*, which thrive in nutrient-poor soils, are significantly abundant in P-deficient conditions, indicating the state of soil P deficiency (Wang et al., 2019).

The activation of soil P is largely dependent on microbial activity, which involves the release of organic and inorganic acids by microbes (Zhu et al., 2018) and the hydrolysis of P compounds via phosphatase activity (Chen et al., 2006; Qiu et al., 2011). In the present study, ACP, Ure, SuCr, and CAT exhibited distinct responses across plantation-age classes and aggregate sizes (Figure 6), indicating shifts in soil biochemical functioning. Under conditions of low P availability, soil inorganic pyrophosphate remains relatively stable (Gutiérrez-Luna et al., 2018). Both microbes and plants can extract inorganic pyrophosphate from less soluble P sources, such as Ca-P, and release significant amounts of H^+^ into the soil (Akhtar et al., 2009). Soil P deficiency was accompanied by shifts in bacterial communities, which, together with changes in phosphatase activity, were associated with the redistribution of relatively refractory P fractions (Figure 10).

**FIGURE 10:**
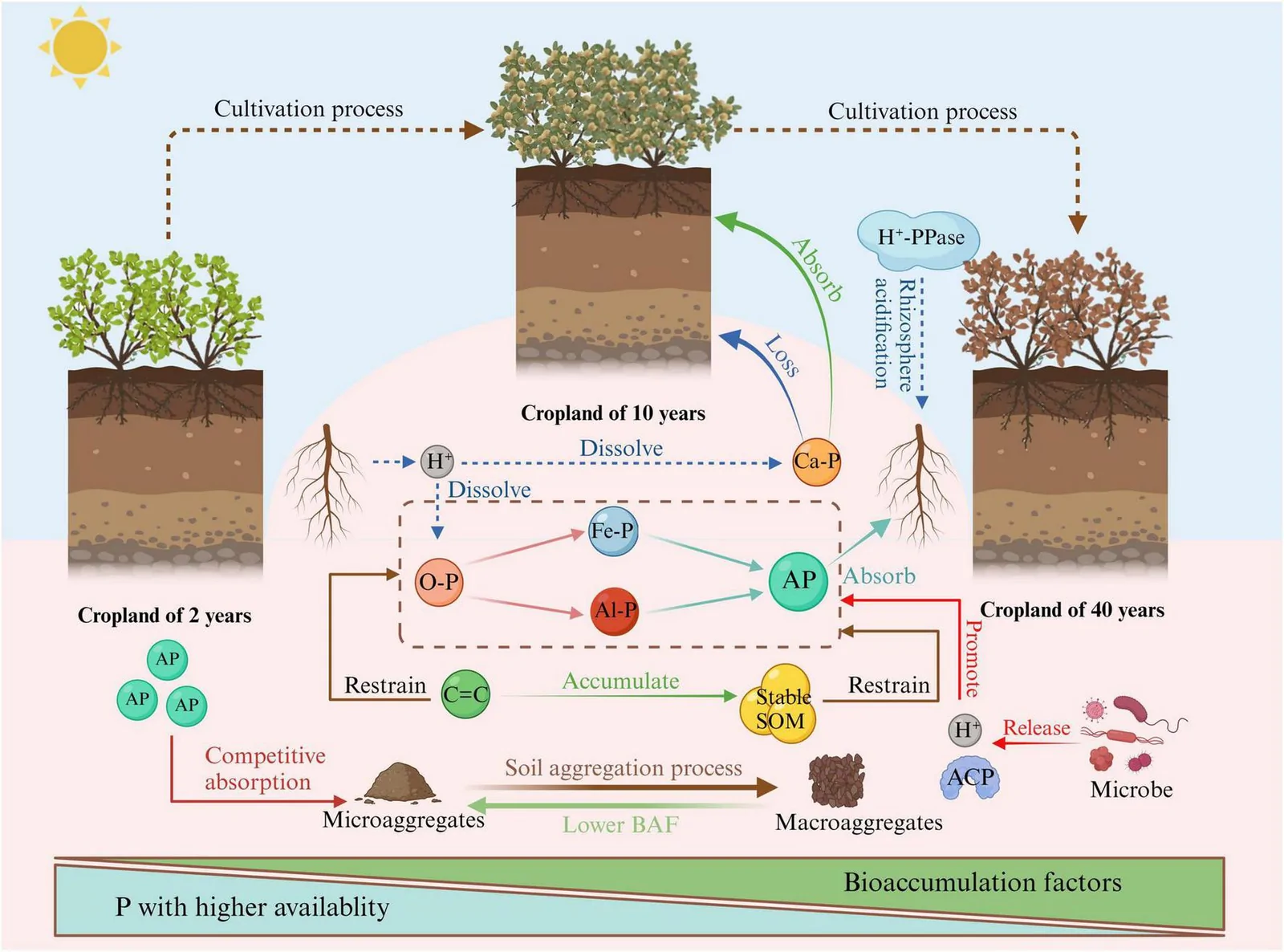
Conceptual diagram illustrating how soil aggregates and their biochemical factors influence the redistribution of soil P fractions and the uptake of AP by *C. oleifera* at different cultivation stand ages (Created with BioRender.com).

However, in this research, several limitations should be considered when interpreting these results. First, the study used a space for time substitution with three independent plots per plantation age class. Although the selected plantations were located within a relatively small geographical area and had similar soil type, initial fertility, topography, cultivar, and management history, residual differences in site history cannot be completely separated from plantation-age effects. The relatively limited number of independent plots (*n* = 3 per plantation age class) also constrains statistical power and may reduce the ability to detect smaller age or aggregate specific effects. Second, bacterial communities were characterized using 16S rRNA gene amplicon sequencing; therefore, the observed taxonomic associations with P fractions do not establish taxon specific P cycling functions. Measurements of functional genes or metagenomic approaches would be required to directly evaluate microbial mechanisms of P transformation. Finally, the sequential extraction procedure provides operationally defined Al, Fe, occluded and Ca associated P pools rather than discrete mineralogical P species. Although comparable fractionation procedures have been applied to acidic, highly weathered red soils, extract selectivity may still be influenced by the Fe/Al-rich mineralogy of allitic soils. Future studies combining sequential extraction with spectroscopic identification of P species would provide stronger mineralogical constraints on P partitioning.

## Conclusion

Soil aggregates provides an important spatial framework for understanding variation in P availability and bacterial communities among *C. oleifera* plantation ages. The marked decline in soil AP was accompanied by greater P adsorption in larger aggregates and aggregate specific redistribution of inorganic P fractions, while Fe-P remained quantitatively dominant. Bacterial community composition differed significantly among plantation ages, while bacterial α-diversity responses varied among aggregate fractions and diversity indices, with O-P, Al-P and Fe-P showing positive associations with bacterial diversity in LM but predominantly negative associations in SM and Mi. Several bacterial groups, including *Proteobacteria*, *Chloroflexi*, *Verrucomicrobia* and *Patescibacteria*, also responded differently to specific P fractions. These findings demonstrate that soil aggregates mediate the linkage between P redistribution and bacterial community assembly during prolonged *C. oleifera* cultivation. This study improves our understanding of the interactions among soil structure, bacterial communities, and P cycling in long-term subtropical plantations. The results also provide a scientific basis for improving PPU and developing P management strategies for aging *C. oleifera* plantations.

## Data Availability

The 16S rRNA sequencing data are available in the NCBI Sequence Read Archive under accession number SRP332322.
