# Supplementary material for: Soil phosphorus partitioning and bacterial communities vary with prolonged Camellia oleifera cultivation
Source: Front Microbiol. 2026 Sep 30;17:1944090. doi: 10.3389/fmicb.2026.1944090 (PMC13661960; doi:10.3389/fmicb.2026.1944090)
Supplement: Supplementary file 1 [file Table_1.docx]

Fig. S1 The linear relationship between various environment factors including pH, acid phosphatase (ACP), available potassium (AK) and available phosphorus (AP) content. The shaded areas around the lines represent the 95% confidence interval. *p<0.05; **p<0.01; ***p<0.001 based on linear regression.

Fig. S2 The decomposability of SOM (CH/COO) in different soil aggregates during various cultivation periods. Statistical significance among the cultivation periods is denoted by different letters (P < 0.05) (a). The linear relationship between SOM and decomposability of SOM (CH\COO) in different soil aggregates (b).

Fig. S3 The linear relationship between soil Ca-P content and soil functional groups in different aggregates. The soil aggregate sizes are categorized as follows: LM, large macroaggregate (>2 mm); SM, small microaggregate (2–0.25 mm); and Mi, microaggregate (<0.25 mm). The shaded areas around the lines represent the 95% confidence interval. *p<0.05; **p<0.01; ***p<0.001 based on linear regression.

Fig. S4 The variations in microbial diversity across three distinct soil aggregates during different cultivation periods, with significant differences among cultivation periods denoted by distinct letters (P < 0.05).

Fig. S5 The linear relationship between soil phosphorus fractions content and microbial indicator (OTUs and Simpsons). *p<0.05; **p<0.01; ***p<0.001 based on linear regression.

Table. S1 The content of major chemical factors in different soil aggregates during various cultivation periods.

| Treatments | | TP (g/kg) | AP (mg/kg) | SOM (g/kg) | pH |
| --- | --- | --- | --- | --- | --- |
| Chi | LM | 0.19 ± 0.05 | 1.25 ± 0.50 | 13.37 ± 0.86 | 4.79 ± 0.12 |
|  | SM | 0.19 ± 0.05 | 1.23 ± 0.36 | 14.16 ± 0.82 | 4.58 ± 0.17 |
|  | Mi | 0.18 ± 0.06 | 1.40 ± 0.40 | 14.30 ± 0.49 | 4.70 ± 0.08 |
| You | LM | 0.13 ± 0.01 | 0.74 ± 0.60 | 25.41 ± 4.08 | 4.12 ± 0.28 |
|  | SM | 0.14 ± 0.01 | 0.77 ± 0.49 | 23.14 ± 2.34 | 4.00 ± 0.76 |
|  | Mi | 0.14 ± 0.01 | 0.97 ± 0.49 | 35.20 ± 0.92 | 4.16 ± 0.34 |
| Old | LM | 0.14 ± 0.02 | 0.20 ± 0.08 | 34.36 ± 2.90 | 4.37 ± 0.01 |
|  | SM | 0.15 ± 0.01 | 0.20 ± 0.12 | 36.52 ± 0.63 | 4.28 ± 0.06 |
|  | Mi | 0.14 ± 0.01 | 0.44 ± 0.11 | 55.53 ± 0.82 | 4.37 ± 0.07 |
| Cutivation period (C) | | *** | *** | *** | *** |
| Soil aggregates (S) | | ns | ns | *** | ns |
| C × S | | ns | ns | *** | ns |

Table. S2 Phosphorus adsorption parameters during various cultivation periods in different soil aggregates.

| Treatments | | Q_m_ (mg·kg^-1^) | MBC (mg·kg^-1^) | K_L_ | R^2^ |
| --- | --- | --- | --- | --- | --- |
| Chi | LM | 1716.21 | 1806.54 | 0.95 | 0.93 |
|  | SM | 4362.99 | 1825.52 | 2.39 | 0.95 |
|  | Mi | 3954.94 | 1865.54 | 2.12 | 0.95 |
| You | LM | 2584.49 | 1820.06 | 1.42 | 0.92 |
|  | SM | 1759.69 | 1852.30 | 0.95 | 0.94 |
|  | Mi | 3892.15 | 1745.36 | 2.23 | 0.92 |
| Old | LM | 2306.98 | 1971.78 | 1.17 | 0.92 |
|  | SM | 1755.17 | 1809.45 | 0.97 | 0.94 |
|  | Mi | 3846.69 | 1756.48 | 2.19 | 0.92 |

Note: Maximal adsorption capacity (Q_m_), maximum buffer capacity (MBC), Langmuir adsorption constant (K_L_).

Table. S3 Relative content of SOM soil functional groups in different soil aggregates during various cultivation periods.

| Treatments | | C-O=O (%) | C-H (%) | C=C (%) | O-H (%) | -OH (%) |
| --- | --- | --- | --- | --- | --- | --- |
| Chi | LM | 2.26 ± 0.15 | 4.23 ± 0.15 | 1.20 ± 0.10 | 26.93 ± 0.15 | 23.00 ± 0.26 |
|  | SM | 5.83 ± 0.25 | 1.77 ± 0.15 | 7.93 ± 0.45 | 25.03 ± 1.05 | 16.67 ± 0.60 |
|  | Mi | 4.40 ± 0.26 | 3.66 ± 0.15 | 1.03 ± 0.11 | 27.40 ± 0.65 | 23.30 ± 0.92 |
| You | LM | 3.73 ± 0.15 | 1.47 ± 0.15 | 15.13 ± 0.25 | 28.03 ± 0.21 | 13.90 ± 0.45 |
|  | SM | 6.26 ± 0.55 | 4.67 ± 0.55 | 13.77 ± 0.35 | 4.60 ±  0.20 | 20.33 ± 0.47 |
|  | Mi | 3.73 ± 0.42 | 1.77 ± 0.15 | 13.90 ± 0.55 | 4.83 ±  0.21 | 20.46 ± 1.35 |
| Old | LM | 5.73 ± 0.15 | 1.33 ± 0.15 | 2.67 ± 0.15 | 10.47 ± 0.47 | 18.63 ± 0.30 |
|  | SM | 5.03 ± 0.35 | 2.27 ± 0.21 | 4.00 ± 0.10 | 27.13 ± 0.83 | 16.43 ± 0.61 |
|  | Mi | 2.73 ± 0.32 | 0.53 ± 0.15 | 15.50 ± 0.53 | 18.60 ± 1.34 | 13.30 ± 0.55 |
| Cultivation period (C) | | *** | *** | *** | *** | *** |
| Soil aggregates (S) | | *** | *** | *** | *** | *** |
| C × S | | *** | *** | *** | *** | *** |
